# Research Progress of Puerarin and Its Benefits in Nonalcoholic Fatty Liver Disease: A Bibliometric Analysis

**DOI:** 10.1002/fsn3.71926

**Published:** 2026-05-23

**Authors:** Wei Wang, Xiang Liu

**Affiliations:** ^1^ Department of Clinical Pharmacy Xiangtan Central Hospital (The Affiliated Hospital of Hunan University) Xiangtan China

**Keywords:** bibliometric analysis, fibrosis, NAFLD, NASH, puerarin

## Abstract

With the development of society and the economy, nonalcoholic fatty liver disease (NAFLD) has become a major chronic disease in contemporary society. Finding a safe, effective, and economical method is essential for the treatment of NAFLD. Puerarin is a unique component of pueraria. In recent years, puerarin has gradually become the focus of research in the natural products field. This article analyzes the research trend of puerarin through VOSviewer software and CiteSpace software. This will be conducive to deepening the understanding of puerarin. Meanwhile, this article also explores the toxicity, pharmacokinetics, and pharmacological activity of puerarin, as well as the mechanism and progress of puerarin in the treatment of NAFLD, which also provides valuable references for future research in this field.

## Graphical abstract

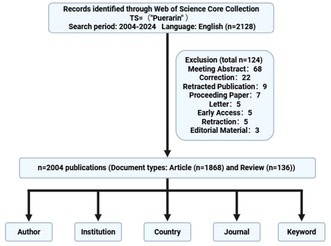

## 1 Introduction

**FIGURE 1:**
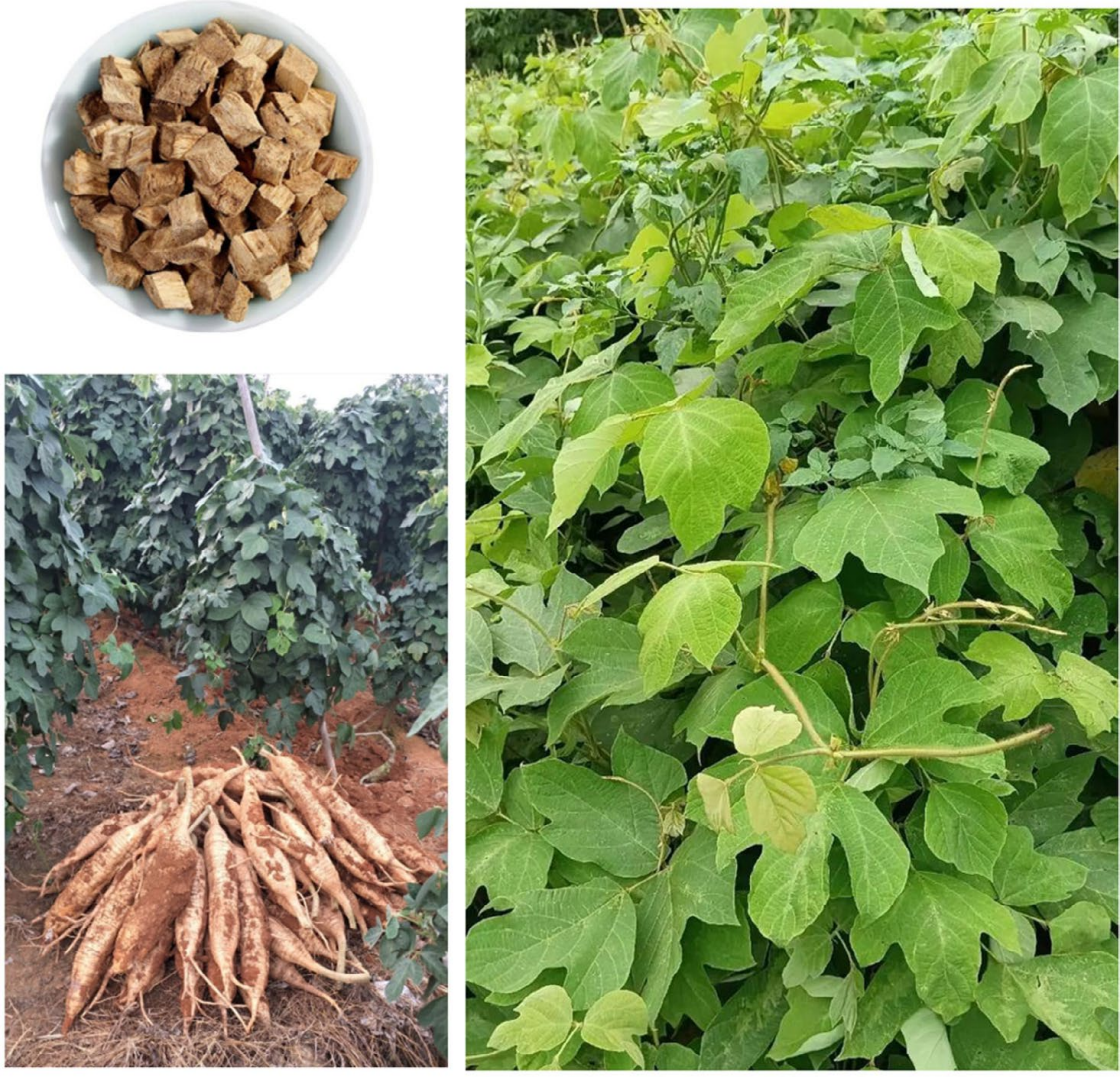
The botany of *Pueraria*.

**FIGURE 2:**
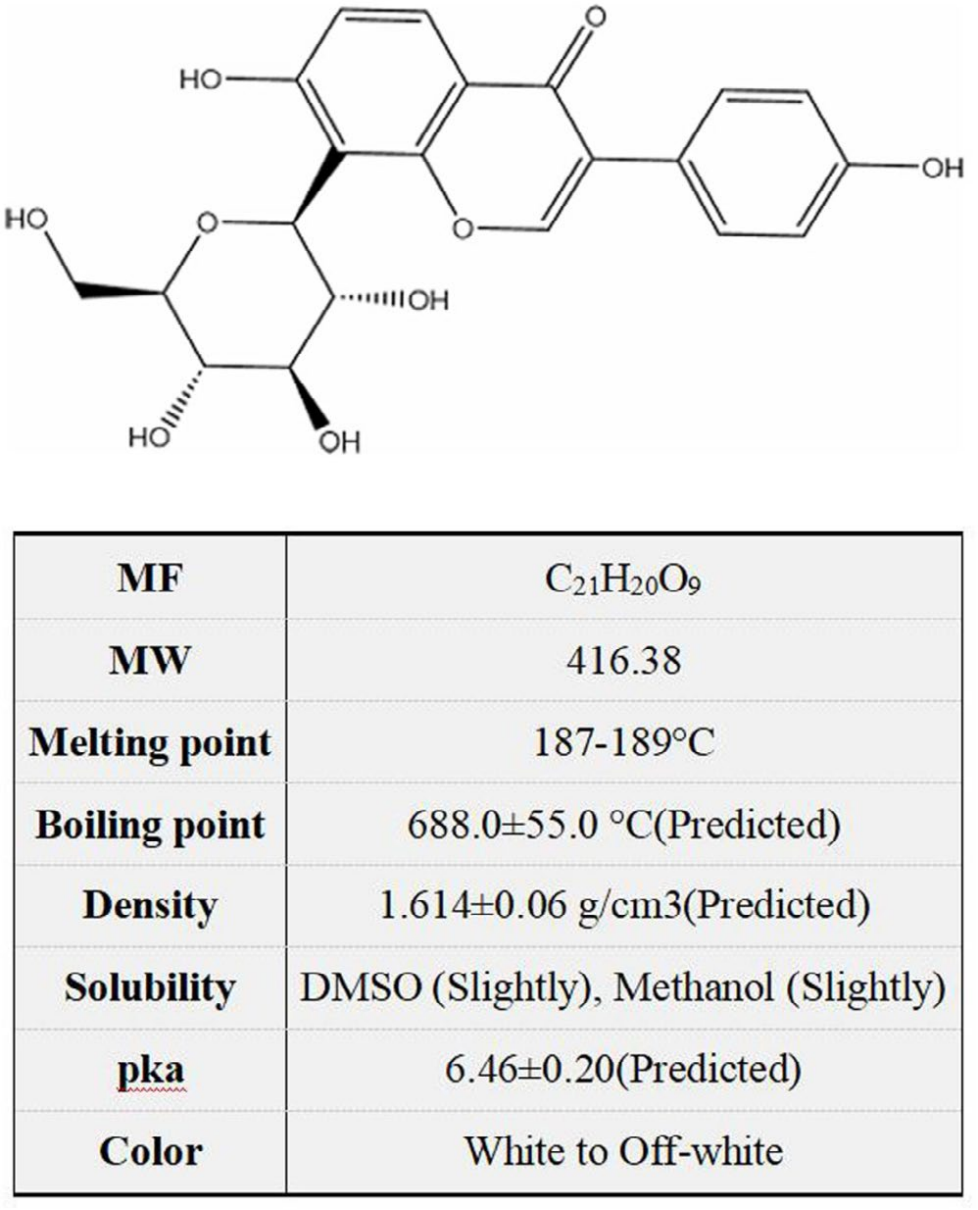
The structure and properties of puerarin.

**FIGURE 3:**
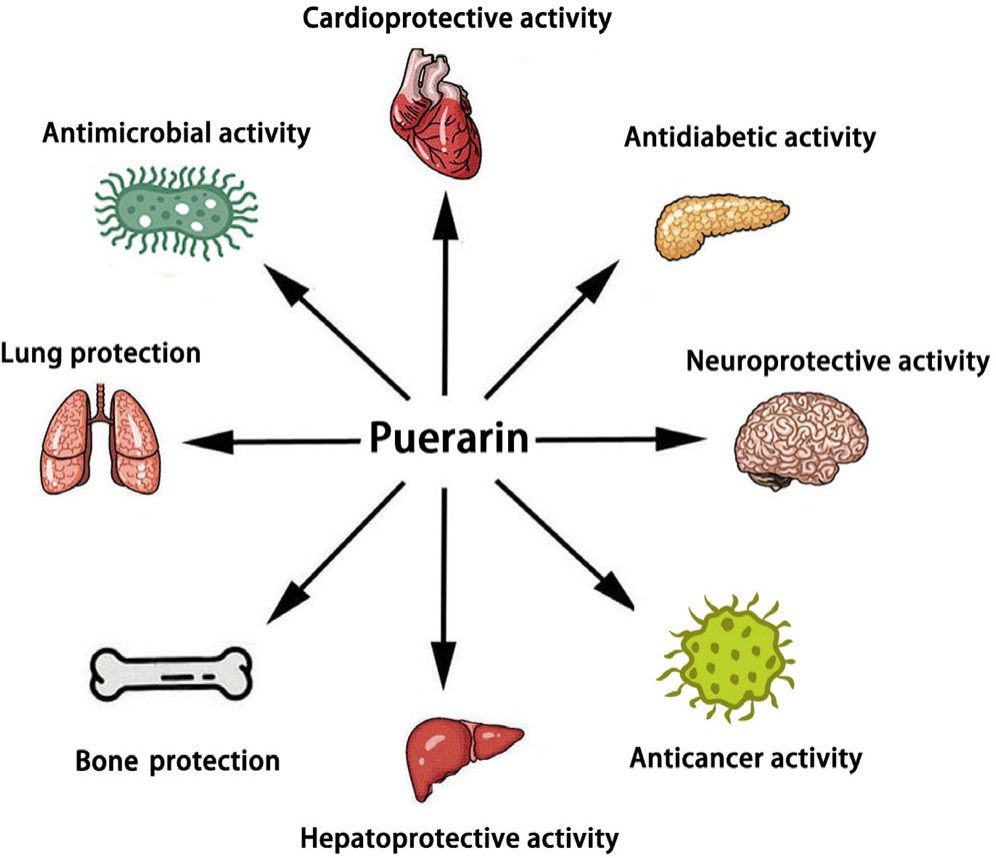
The pharmacological activity of puerarin.

Nonalcoholic fatty liver disease (NAFLD) is a clinicopathologic syndrome characterized by excessive fat deposition in liver cells, which is caused by no‐alcohol and other specific liver damaging factors. Puerarin, a unique component of Pueraria (Figure 1), is also known as “phytoestrogen” (Zhou et al. 2014). Its chemical name is 8‐β‐D‐grape pyranose‐4, 7‐dihydroxyisoflavone; molecular formula is C_21_H_20_O_9_, relative molecular weight is 416.38 (Figure 2). Puerarin is a white needle‐like crystal and soluble in methanol, slightly soluble in ethanol and water (Wang et al. 2022). Contemporary research has demonstrated that the main active components of puerarin are flavonoids, including daidzin, daidzein, puerarin, allantoin, β‐sitosterol, starch, etc. (Wei et al. 2014). The extraction of puerarin is the key to analyzing its structure and biological properties. Therefore, it is very important to investigate the extraction technology of puerarin. At present, the extraction methods of puerarin mainly include solvent extraction, ultrasound extraction, enzyme‐assisted extraction, and microwave‐assisted extraction (Liga and Paul 2024). In addition, the detection methods of puerarin mainly include liquid chromatography, high‐performance liquid chromatography, capillary electrophoresis, and ultraviolet spectroscopy (Liu et al. 2012; Ma et al. 2005). However, due to the time‐consuming nature, expensive instruments, and high experimental costs, the application of these methods was always limited in real sample measurements. Puerarin has been widely used in the treatment of neurological disease, cardiovascular disease, cancer, virus infection, bone damage, diabetes, colitis, autoimmune thyroiditis, chronic pancreatitis, lung injury, irritable bowel syndrome, etc. (Figure 3). In addition to treating diseases, numerous studies have also shown that puerarin plays an important role in agriculture and animal husbandry. On the one hand, puerarin can be used as a feed additive to improve the immunity of broilers, sows, and pigeons (Cao et al. 2024; Wang, Li, et al. 2024; Zhou, Yu, et al. 2023). On the other hand, puerarin can improve the meat quality of beef cattle (Li, Shang, et al. 2020).

## 2 Method

**FIGURE 4:**
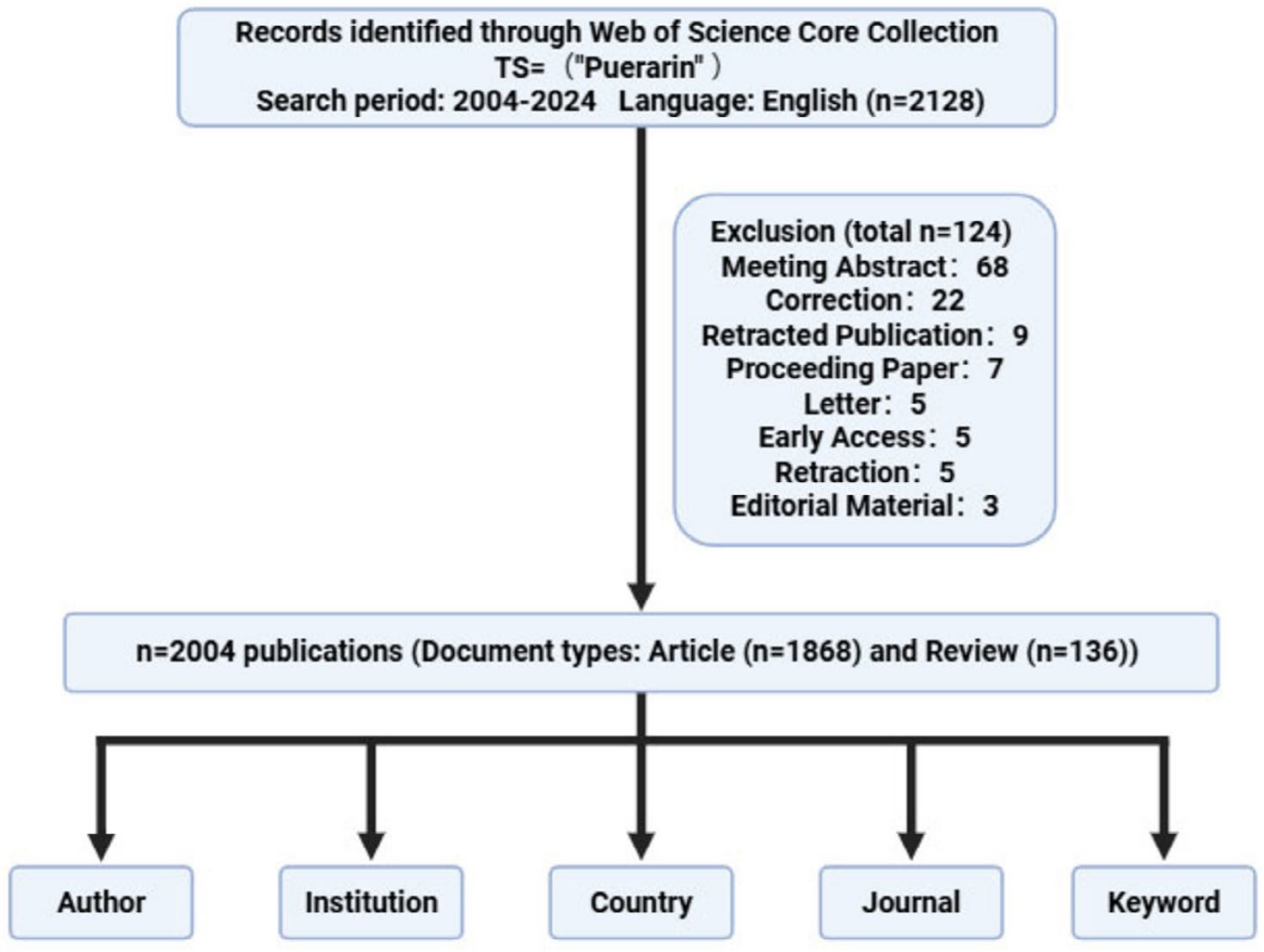
Flow chart of bibliometric analysis.

The search was conducted on June 1, 2025, using the Web of Science Core Collection (WOS SCI‐EXPANDED). The search query was: TS = (“puerarin”) AND DT = (“Article” OR “Review Article”) AND PY = (2004–2024). A total of 2004 papers were included (Figure 4). To ensure reliability, two investigators independently screened titles and abstracts to exclude irrelevant content. The disagreements among them were resolved by the third investigator. Then, the VOSviewer software and CiteSpace software were used to conduct visual analysis and cluster analysis on the relevant content.

## 3 Bibliometric Analysis of Literature

### 3.1 General Analysis

**FIGURE 5:**
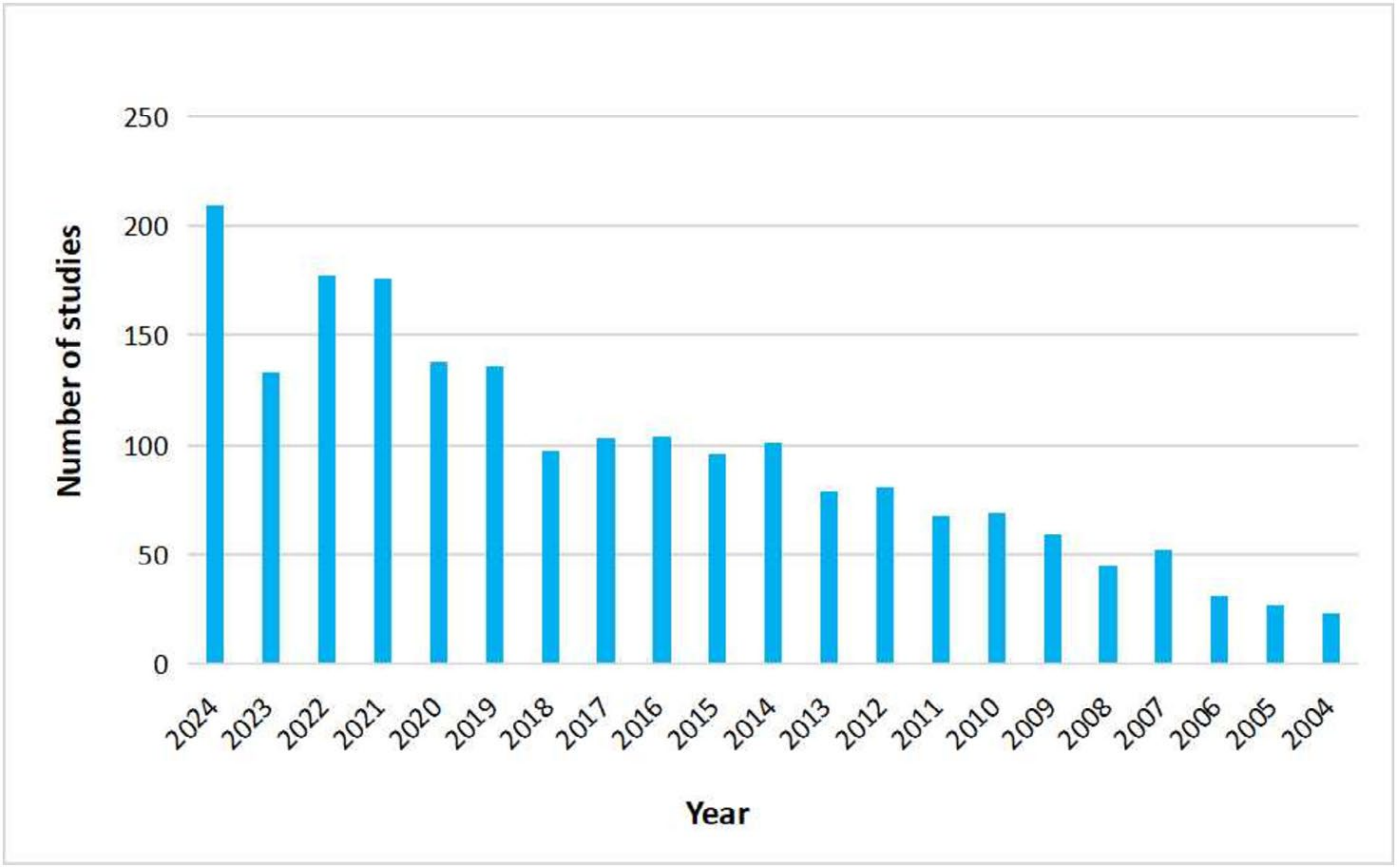
Annual publication count for puerarin research from 2004 to 2024.

The analysis of publication trends can offer insights into the growth and scope of the research field. In this section, a total of 2004 articles were identified. As shown in Figure 5, our dataset reveals a consistent upward trajectory in puerarin‐related research output. The publication peak was reached in 2024 (209 articles). To explore the authors, institutions, countries, and journals in the fields of puerarin, co‐authorship networks were visualized using VOSviewer software.

### 3.2 Author Analysis

**FIGURE 6:**
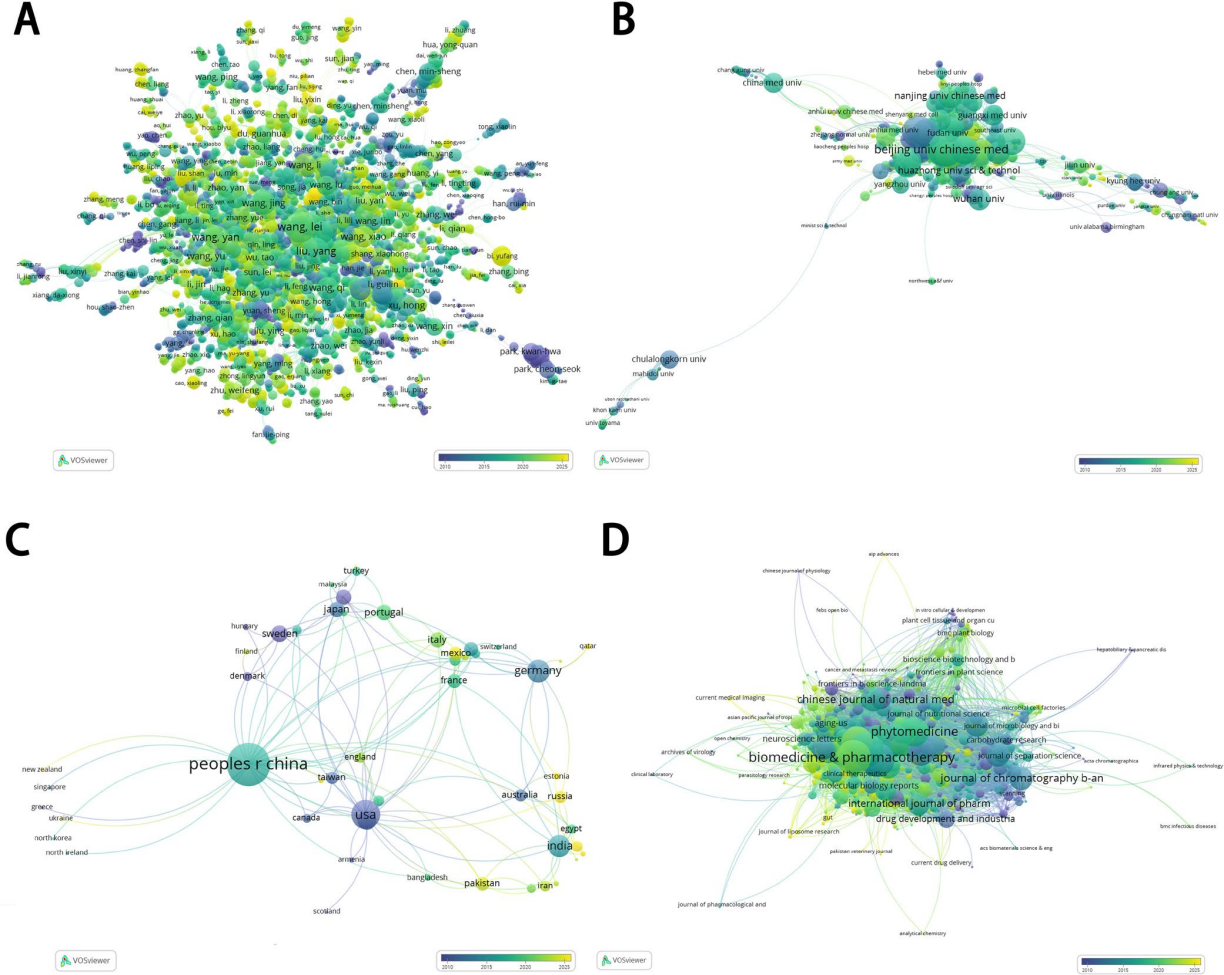
(A) Visualization of author network. (B) Visualization of institution network. (C) Visualization of the country network. (D) Visualization of journal network.

The 2004 articles include 9579 authors. As shown in Figure 6A and Table 1, Wang, Lei (21 articles) published the largest number of articles, followed by Liu, Yang (16 articles), Wang, Yan (16 articles), Tan, Tianwei (15 articles), Liang, Tao (13 articles), Lu, Yang (13 articles), Wang, Jing (13 articles), Wang, Li (13 articles), Wang, Qi (13 articles), and Zhang, Jing (13 articles). Wang, Lei (180) had the closest cooperation, followed by Liang, Shangdong (122), Liu, Shuangmei (119), Gao, Yun (118), Liu, Yang (113), Li, Guilin (109), Wang, Li (101), Chen, Min‐sheng (101), Wang, Yan (97), and Xu, Hong (95).

**TABLE 1:** Top 10 authors in puerarin research.

Rank	Author	Number of studies	Rank	Author	Total link strength
1	Wang, Lei	21 articles	1	Wang, Lei	180
2	Liu, Yang	16 articles	2	Liang, Shangdong	122
3	Wang, Yan	16 articles	3	Liu, Shuangmei	119
4	Tan, Tianwei	15 articles	4	Gao, Yun	118
5	Liang, Tao	13 articles	5	Liu, Yang	113
6	Lu, Yang	13 articles	6	Li, Guilin	109
7	Wang, Jing	13 articles	7	Wang, Li	101
8	Wang, Li	13 articles	8	Chen, Min‐sheng	101
9	Wang, Qi	13 articles	9	Wang, Yan	97
10	Zhang, Jing	13 articles	10	Xu, Hong	95

### 3.3 Institution Analysis

As shown in Figure 6B and Table 2, Beijing University of Chinese Medicine has the largest number of published papers. University of Chinese Academy of Sciences has the closest cooperation. The 2004 articles cover 1721 institutions. The top 10 organizations which have published related articles are Beijing University of Chinese Medicine (62 articles), Zhejiang University (56 articles), Shandong University (45 articles), Shenyang Pharmaceutical University (45 articles), Nanchang University (42 articles), University of Chinese Academy of Sciences (41 articles), Guangzhou University of Chinese Medicine (40 articles), Chengdu University of Traditional Chinese Medicine (38 articles), Southern Medical University (36 articles), and Capital Medical University (33 articles). According to total link strength, the top 10 organizations are University of Chinese Academy of Sciences (92), Guangzhou University of Chinese Medicine (88), Zhejiang University (71), Southern Medical University (69), Beijing University of Chinese Medicine (66), Capital Medical University (64), China Academy of Chinese Medical Sciences (64), Shandong University (62), Guangzhou Medical University (61), and Nanjing University of Chinese Medicine (59).

**TABLE 2:** Top 10 institutions publishing puerarin studies.

Rank	Institution	Number of studies	Rank	Institution	Total link strength
1	Beijing University of Chinese Medicine	62 articles	1	University of Chinese Academy of Sciences	92
2	Zhejiang University	56 articles	2	Guangzhou University of Chinese Medicine	88
3	Shandong University	45 articles	3	Zhejiang University	71
4	Shenyang Pharmaceutical University	45 articles	4	Southern Medical University	69
5	Nanchang University	42 articles	5	Beijing University of Chinese Medicine	66
6	University of Chinese Academy of Sciences	41 articles	6	Capital Medical University	64
7	Guangzhou University of Chinese Medicine	40 articles	7	China Academy of Chinese Medical Sciences	64
8	Chengdu University of Traditional Chinese Medicine	38 articles	8	Shandong University	62
9	Southern Medical University	36 articles	9	Guangzhou Medical University	61
10	Capital Medical University	33 articles	10	Nanjing University of Chinese Medicine	59

### 3.4 Country Analysis

The 2004 articles include 63 countries. As shown in Figure 6C and Table 3, China (1670 articles) published the largest number of articles, followed by the USA (116 articles), South Korea (90 articles), Japan (40 articles), India (39 articles), China Taiwan (34 articles), Thailand (32 articles), Germany (24 articles), Australia (18 articles), and Sweden (14 articles). China (158) had the closest cooperation, followed by the USA (95), South Korea (33), Germany (23), Japan (21), Sweden (19), India (18), Thailand (16), Australia (16), and Denmark (14).

**TABLE 3:** Top 10 countries publishing puerarin studies.

Rank	Country	Number of studies	Rank	Country	Total link strength
1	China	1670 articles	1	China	158
2	USA	116 articles	2	USA	95
3	South Korea	90 articles	3	South Korea	33
4	Japan	40 articles	4	Germany	23
5	India	39 articles	5	Japan	21
6	China Taiwan	34 articles	6	Sweden	19
7	Thailand	32 articles	7	India	18
8	Germany	24 articles	8	Thailand	16
9	Australia	18 articles	9	Australia	16
10	Sweden	14 articles	10	Denmark	14

### 3.5 Journal Analysis

As shown in Figure 6D and Table 4, Journal of ethnopharmacology has the largest number of published papers, and Biomedicine & pharmacotherapy has the closest cooperation. The 2004 articles are from 635 kinds of journals. The top 10 journals that have published related articles are Journal of ethnopharmacology (50 articles), Frontiers in pharmacology (49 articles), Journal of Chromatography B‐Analytical Technologies in the Biomedical and Life Sciences (40 articles), Molecules (38 articles), Biomedicine & pharmacotherapy (36 articles), Journal of pharmaceutical and biomedical analysis (35 articles), Phytomedicine (32 articles), Biomedical chromatography (30 articles), Journal of agricultural and food chemistry (26 articles), and Molecular medicine reports (25 articles). According to total link strength, the top 10 journals are Biomedicine & pharmacotherapy (913), Frontiers in pharmacology (726), Journal of ethnopharmacology (695), European journal of pharmacology (683), Phytotherapy research (667), Phytomedicine (535), Journal of Chromatography B‐Analytical Technologies in the Biomedical and Life Sciences (448), American journal of chinese medicine (398), Molecular medicine reports (388), and Journal of agricultural and food chemistry (374).

**TABLE 4:** Top 10 journals publishing puerarin studies.

Rank	Journal	Number of studies	Rank	Journal	Total link strength
1	Journal of ethnopharmacology	50 articles	1	Biomedicine & pharmacotherapy	913
2	Frontiers in pharmacology	49 articles	2	Frontiers in pharmacology	726
3	Journal of Chromatography B‐Analytical Technologies in the Biomedical and Life Sciences	40 articles	3	Journal of ethnopharmacology	695
4	Molecules	38 articles	4	European journal of pharmacology	683
5	Biomedicine & pharmacotherapy	36 articles	5	Phytotherapy research	667
6	Journal of pharmaceutical and biomedical analysis	35 articles	6	Phytomedicine	535
7	Phytomedicine	32 articles	7	Journal of Chromatography B‐Analytical Technologies in the Biomedical and Life Sciences	448
8	Biomedical chromatography	30 articles	8	American journal of chinese medicine	398
9	Journal of agricultural and food chemistry	26 articles	9	Molecular medicine reports	388
10	Molecular medicine reports	25 articles	10	Journal of agricultural and food chemistry	374

### 3.6 Keyword Analysis

**FIGURE 7:**
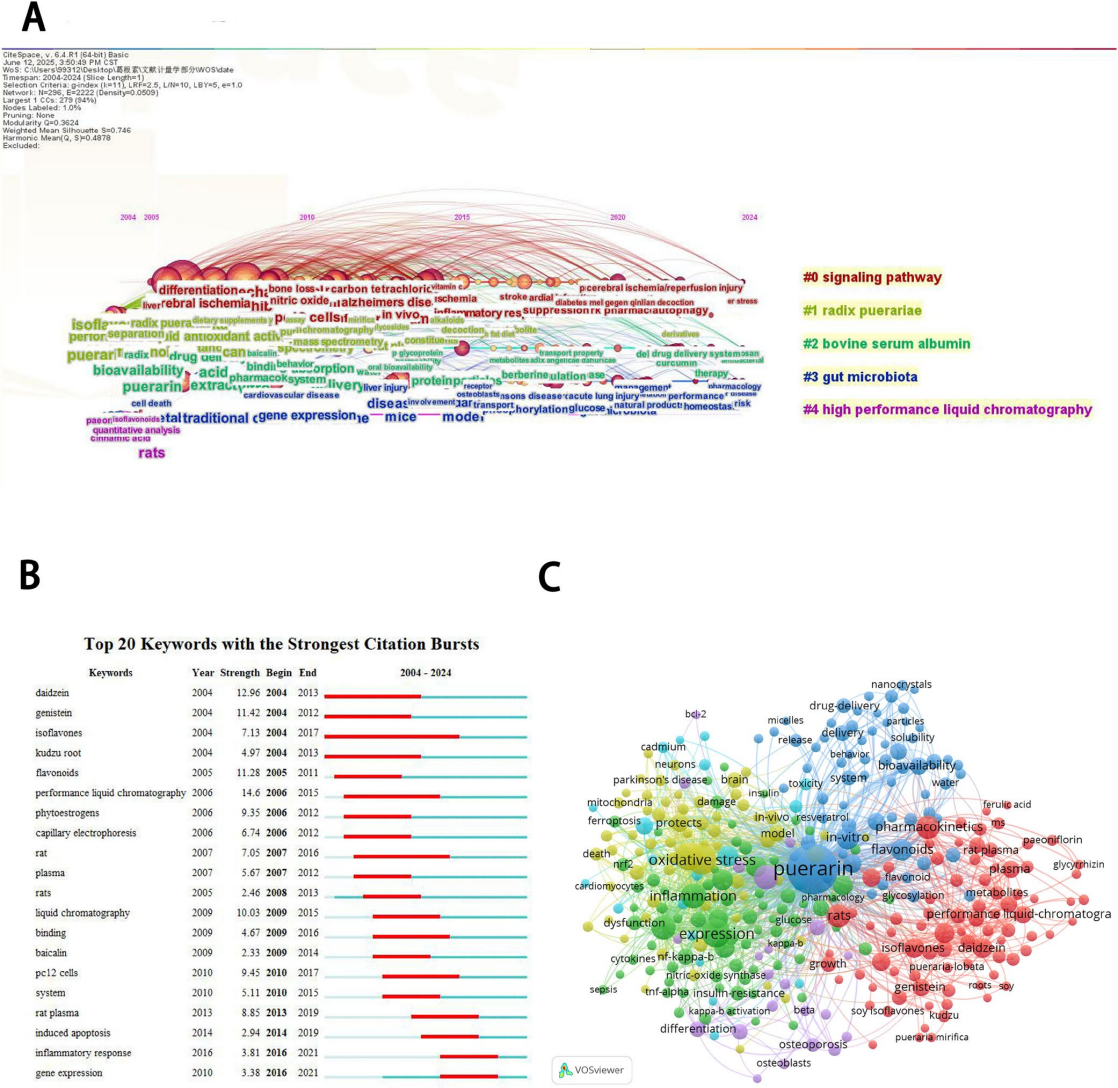
(A) Timeline view of keyword clusters. (B) Top 20 keywords with the strongest citation bursts. (C) Visualization of the keyword network.

This article identified 7901 keywords through retrieval. The co‐occurrence analysis of keywords was conducted through VOS software. As shown in Figure 7A, the keywords are divided into 5 clusters: Cluster #0 (signaling pathway), Cluster #1 (radix puerariae), Cluster #2 (bovine serum albumin), Cluster #3 (gut microbiota), Cluster #4 (high performance liquid chromatography). As shown in Figure 7C, the top 10 high‐frequency keywords are puerarin (1243), oxidative stress (266), expression (240), apoptosis (235), inflammation (168), activation (157), cells (155), pharmacokinetics (142), rats (140), in vitro (128). According to total link strength, the top 10 keywords are puerarin (13708), oxidative stress (3228), expression (2801), apoptosis (2708), inflammation (1964), activation (1929), cells (1774), in vitro (1637), rats (1621), pharmacokinetics (1530). Emergent words are keywords that experience a significant increase within a certain period. This study utilized CiteSpace to perform an emergent words analysis and generate an emergent map. As shown in Figure 7B, the burst strengths range from 2.33 to 12.96.

## 4 Toxicity

It is well known that the side effects of natural medicines often limit their clinical application. Therefore, understanding the side effects of natural medicines is of great significance for the improvement and application of natural medicines in the future. Xie XS evaluated the safety of puerarin injection in clinical use (Xie et al. 2018). They collected and analyzed 62 papers related to adverse drug reactions of puerarin injection. The results showed that adverse drug reactions of puerarin injection occurred mostly in patients aged 50–79 years and the immune/blood system accounted for the majority. Puerarin also induced some relatively uncommon diseases (drug‐induced immune hemolytic anemia) (Chen et al. 2013). Hemolysis largely limited the clinical use of puerarin injections. Hou SZ investigated the mechanisms of puerarin‐induced hemolysis (Hou et al. 2011). They found that high concentrations of puerarin‐induced hemolysis were linked to the changes in membrane lipids and erythrocyte membrane proteins. It is worth noting that different preparations will significantly improve the adverse drug reactions of puerarin. Yue PF studied a submicron emulsion of puerarin by a novel complex‐phase inversion‐high press homogenization technology (Yue et al. 2008). They found that submicron emulsions significantly reduced the hemolysis of puerarin. In addition to hemolysis, puerarin may have teratogenic risk. Huang FJ found that puerarin has deleterious effects on mouse oocyte maturation and embryonic development (Huang and Chan 2016). Interestingly, some studies have suggested that puerarin may ameliorate male fertility impairment (Li et al. 2023). As an important oxidase family, CYP enzymes are widely present in animals, plants, microorganisms, and humans. One of its important functions is to participate in the biological transformation of many drugs. Puerarin affected the activities of CYP2B1, CYP2E1, CYP1A2, and CYP2D6, but it had little effect on CYP3A4 activity. This also provides a potential reference for the safety of clinical drug use (Zheng et al. 2010). In conclusion, clinicians should pay attention to the hemolysis, teratogenicity and CYP interactions of puerarin. Due to multiple diseases, reduced liver and kidney functions, and the use of various medications, the elderly are prone to hemolytic reactions and CYP interactions. The teratogenic risk suggests that puerarin should be used with caution in pregnant or pre‐pregnant women. Regarding the toxicity of puerarin, there are still many issues to be addressed (Are the metabolites of puerarin more hemolytic than puerarin itself? Is the effect of puerarin on oocytes reversible? Does the influence of puerarin on CYP enzymes have a circadian rhythm or gender difference?). At present, there are relatively few studies on the toxicity of puerarin. In the future, researchers can improve and understand the toxicity research from the following aspects: (1) Optimizing the preparation process of the puerarin to reduce the toxicity of the drugs. (2) Strengthening the monitoring of adverse reactions of the puerarin and encourage the conduct of large‐scale, multi‐center, and prospective safety studies. (3) Utilizing new technologies to evaluate the safety of puerarin in models that are closer to human physiology.

## 5 Pharmacokinetics

In recent years, drug delivery systems that enhance the stability of puerarin have become a hot topic. Therefore, it is very important to understand the pharmacokinetics of puerarin. According to the category of the biopharmaceutics classification system, puerarin could be categorized IV drug (low solubility and low permeability value) (Li et al. 2014). Yang R investigated the pharmacokinetics of puerarin in rats by flow‐injection chemiluminescence (Yang et al. 2011). They found that the pharmacokinetics of puerarin were consistent with a two‐compartment open model. The area under the concentration‐time curve, Mean Residence Time, and maximum concentration were 56.67 ± 10.65 mg**·**h/L, 5.04 ± 2.78 h, and 19.70 ± 4.67 μg/mL, respectively. Anukunwithaya T also investigated the pharmacokinetics of puerarin in rats (Anukunwithaya et al. 2018). They found that the bioavailability of puerarin (5 mg/kg and 10 mg/kg orally) was approximately 7%. After intravenous administration, puerarin was widely distributed in the heart, lungs, stomach, liver, kidney, spleen, and brain. As the major metabolites of puerarin, glucuronides were mainly excreted in the urine. It is worth noting that the concentration‐time curve (219.83 ± 64.37 to 462.62 ± 51.74 μg**·**h/L), half‐time (1.60 ± 0.38 to 12.04 ± 5.10 h), maximum concentration (101.64 ± 41.82 to 48.64 ± 21.47 ng/mL) of *Pueraria lobata* extract may be dramatically different from pure puerarin in the plasma of rat (Zhang et al. 2020). Several studies have shown that gestation influenced the pharmacokinetics of puerarin during the early stages of pregnancy (Cao et al. 2013). Furthermore, puerarin penetrates the placental barrier and maintains high concentrations in fetal rat plasma. In addition, many drugs can affect the pharmacokinetics of puerarin. For example, glycyrrhizin significantly increased the maximum concentration of puerarin (from 761.25 ± 52.34 to 456.32 ± 34.75 ng/mL), and decreased the area under the concentration‐time curve of puerarin (from 4142.15 ± 558.51 to 2503.74 ± 447.57 μg·h/L) (Zhao et al. 2018). Verapamil significantly increased the maximum concentration of puerarin (from 683.7 ± 51.2 to 933.5 ± 75.8 ng/mL), and decreased the area under the concentration‐time curve of puerarin (from 3687.3 ± 444.6 to 5006.1 ± 658.6 μg·h/L) (Zhou, Song, and Dong, et al. 2019). Astragaloside IV significantly increased the maximum concentration of puerarin (from 760 to 467 ng/mL), and decreased the area under the concentration‐time curve of puerarin (from 4097 to 2330 μg·h/L). Interestingly, puerarin also affects the pharmacokinetics of other drugs (triptolide, baicalin, and warfarin) (Kong et al. 2018; Li et al. 2014; Wang, Wu, et al. 2019). Owing to the poor water solubility and bioavailability of puerarin, the development of puerarin preparations is worth our consideration. Currently, new puerarin preparations include hydrogel, microspheres, nanoparticles, and long‐circulating liposomes (Long et al. 2024; Pan et al. 2023; Wang, Hang, et al. 2024; Zhang et al. 2024). These preparations usually have good solubility, flowability, stability, and low toxicity. Meanwhile, some excellent drug carriers that can improve the bioavailability of puerarin, such as polyamidomine dendrimer, microemulsion vehicle, and ionic liquid, have also been developed (Gu et al. 2013; Wu et al. 2009; Yuan et al. 2022). This will help puerarin to exert pharmacological effects.

## 6 Pharmacological Activity

At present, numerous studies have shown that puerarin plays a beneficial role in neurological diseases (Xiong et al. 2019), cardiovascular diseases (Gao et al. 2022), cancer (Zhang et al. 2014), osteoporosis (Li et al. 2022), diabetes (Chen et al. 2024), colitis (Tao, Liang, et al. 2024), autoimmune thyroiditis (Tao, Chen, et al. 2024), chronic pancreatitis (Zeng et al. 2021), lung injury (Wang et al. 2018), and irritable bowel syndrome (Wang et al. 2021). Puerarin demonstrates potential in multiple disease models, yet its clinical translation is constrained by low bioavailability and formulation challenges.

The research papers on the pharmacological effects of puerarin have shown a significant upward trend. Early studies mainly focused on the beneficial effects of puerarin in cardiovascular diseases, neurological disorders, and tumors. In recent years, the research has mainly concentrated on the beneficial effects of puerarin in metabolic diseases, bone injury, and regulation of the intestinal flora. The emerging hotspots of the pharmacological effects of puerarin mainly include mechanism research centered on signal pathway analysis, as well as the interaction patterns between puerarin and receptors. Currently, although the pharmacological activities of puerarin have been widely reported, the direct molecular targets of its action remain unclear. Additionally, the pharmacological research of puerarin is still mainly conducted through cell experiments and animal models, and there is still a lack of high‐quality clinical trial evidence. Therefore, the future research direction of puerarin should focus on the study of molecular targets and the conduct of rigorous randomized controlled trials.

## 7 Puerarin and NAFLD

### 7.1 Puerarin and Lipid Metabolism Disorders

**FIGURE 8:**
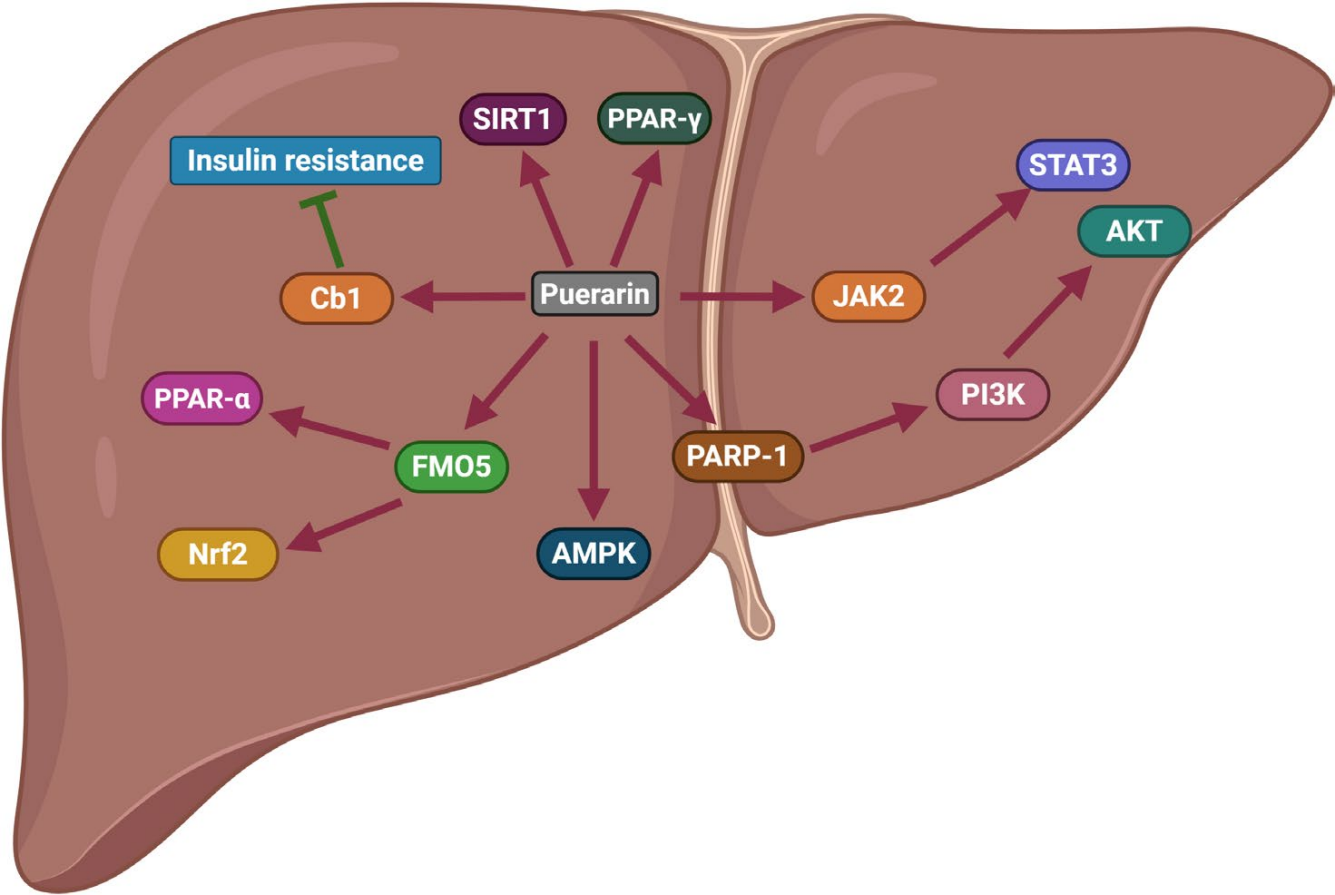
The role of puerarin in regulating lipid metabolism and lipid synthesis.

A large number of studies have shown that puerarin plays an important role in regulating lipid metabolism and lipid synthesis (Figure 8). In 3 T3‐L1 cells and bovine preadipocytes, puerarin regulated adipocyte differentiation (Lee et al. 2010; Yun et al. 2020). In RAW264.7 cells, primary bone marrow‐derived macrophages, and peritoneal macrophages, puerarin dramatically inhibited lipid uptake (Li et al. 2021). In obese mice, puerarin significantly improved free fatty acids, triglycerides, total cholesterol, and LDL‐C (Noh et al. 2022). In Bisphenol S‐induced mice, puerarin reduces lipid synthesis and promotes lipid metabolism by down‐regulating the levels of genes and proteins involved in lipid synthesis (PPARγ, SREBP1C, and FASN) and metabolism (Cpt1a, Cpt1b, and PPARα) (Noh et al. 2022). Lipotoxicity is the accumulation of lipids in non‐fatty tissues (liver, heart, kidneys, muscles, and pancreas) and further damage to these organs or systems (Schaffer 2016). Current studies show that lipid toxicity can drive the occurrence and development of NAFLD by triggering liver endoplasmic reticulum stress and inflammation (He et al. 2024). As an inhibitor of lipid toxicity, puerarin can ameliorate hepatic steatosis of NAFLD mice through the PPARγ signaling pathway, PPAR alpha signaling pathway, SIRT1 signaling pathway, FMO5 signaling pathway, AMPK signaling pathway, and PARP‐1/PI3K/AKT signaling pathway (Kang et al. 2015; Li et al. 2024; Pham et al. 2022; Wang, Yang, et al. 2019, 2014). In NAFLD rats, puerarin ameliorated steatosis and inflammation by modulating the JAK2/STAT3 signaling pathway (Zheng et al. 2009). Insulin resistance is considered to be a key feature of NAFLD. In the state of insulin resistance, increased lipolysis leads to fat accumulation in the liver. Zhao Y found that puerarin improved the insulin resistance of 3 T3‐L1 lipocytes by activating the Cb1 binding protein (Zhao and Zhou 2012). Zhang W found that puerarin improved insulin resistance in HFD‐induced rats (Zhang et al. 2010).

### 7.2 Inflammation

**FIGURE 9:**
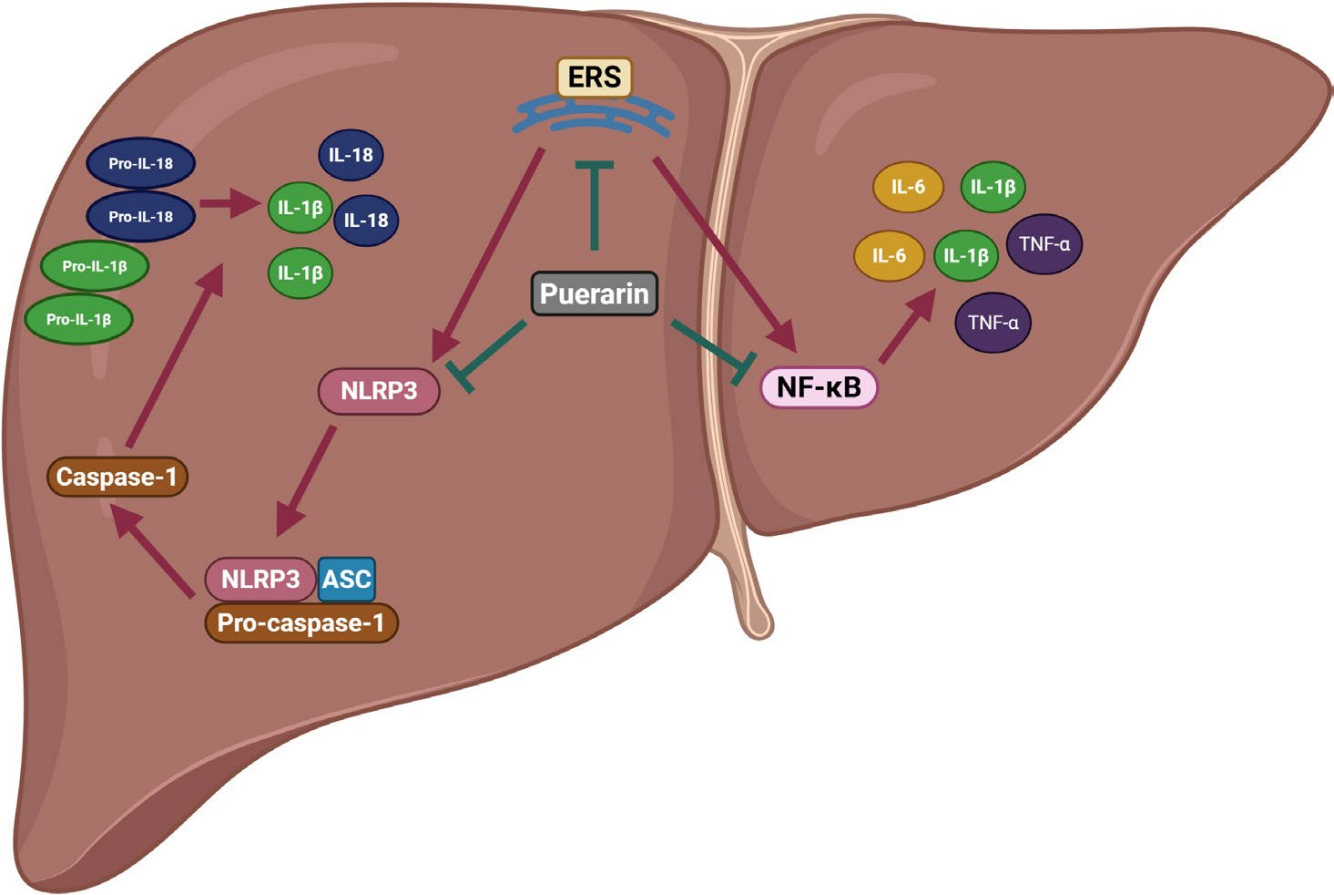
The role of puerarin in regulating inflammation.

As an important driver, inflammation plays an important role in the pathogenesis of NAFLD. Increasing evidence indicates that puerarin efficiently alleviates the progression of NAFLD by suppressing inflammation (Figure 9). Yang M wants to assess the therapeutic effect of puerarin on NAFLD. They found that puerarin reduced the levels of tumor necrosis factor‐alpha (TNF‐α), Interleukin 1β (IL‐1β), and Interleukin 6 (IL‐6) in NAFLD mice. Puerarin ameliorated NAFLD by inhibiting inflammatory cytokines (Yang et al. 2023). Zhou J also assesses the therapeutic effect of puerarin on NAFLD. They found that puerarin ameliorated NAFLD by reducing the levels of TNF‐α, IL‐1β, and Interleukin 18 (IL‐18) (Zhou et al. 2022). Inflammasome is a multiprotein complex in the cytoplasm. Meanwhile, it is also an important component of the immune system. As the most studied inflammasome, NLRP3 inflammasome is composed of NLRP3, apoptosis‐associated blotch‐like protein (ASC), and Caspase‐1. Multiple activators can induce the activation of the NLRP3, which then leads to the secretion of inflammatory cytokines IL‐1β and IL‐18. Activation of NLRP3 inflammasome is proven to be related to the pathogenesis of NAFLD (de Carvalho Ribeiro and Szabo 2022; Ramos‐Tovar and Muriel 2023; Wan et al. 2016). Recent studies have shown that puerarin can not only improve the progression of other diseases (myocardial ischemia/reperfusion injury, lung injury, gastritis) by inhibiting NLRP3 (Cai et al. 2022; Peng and Liu 2022; Wang, Chen, et al. 2020), but also liver injury (Yu et al. 2021). Therefore, NLRP3 inflammasome may be a potential target for puerarin to inhibit the development of NAFLD. In addition to the NLRP3 inflammasome, Endoplasmic reticulum stress (ERS) also plays an important role in the pathogenesis of NAFLD. On the one hand, ERS activates the NLRP3 inflammasome through the UPR pathway and non‐UPR pathway (Li, Cao, et al. 2020). On the other hand, ERS induces the activation of the nuclear factor‐κB (NF‐κB) signaling pathway (Kim et al. 2014). Some studies also proved that puerarin plays an anti‐inflammatory role by inhibiting ERS (Sun et al. 2024; Wang et al. 2017).

### 7.3 Other Mechanisms

**FIGURE 10:**
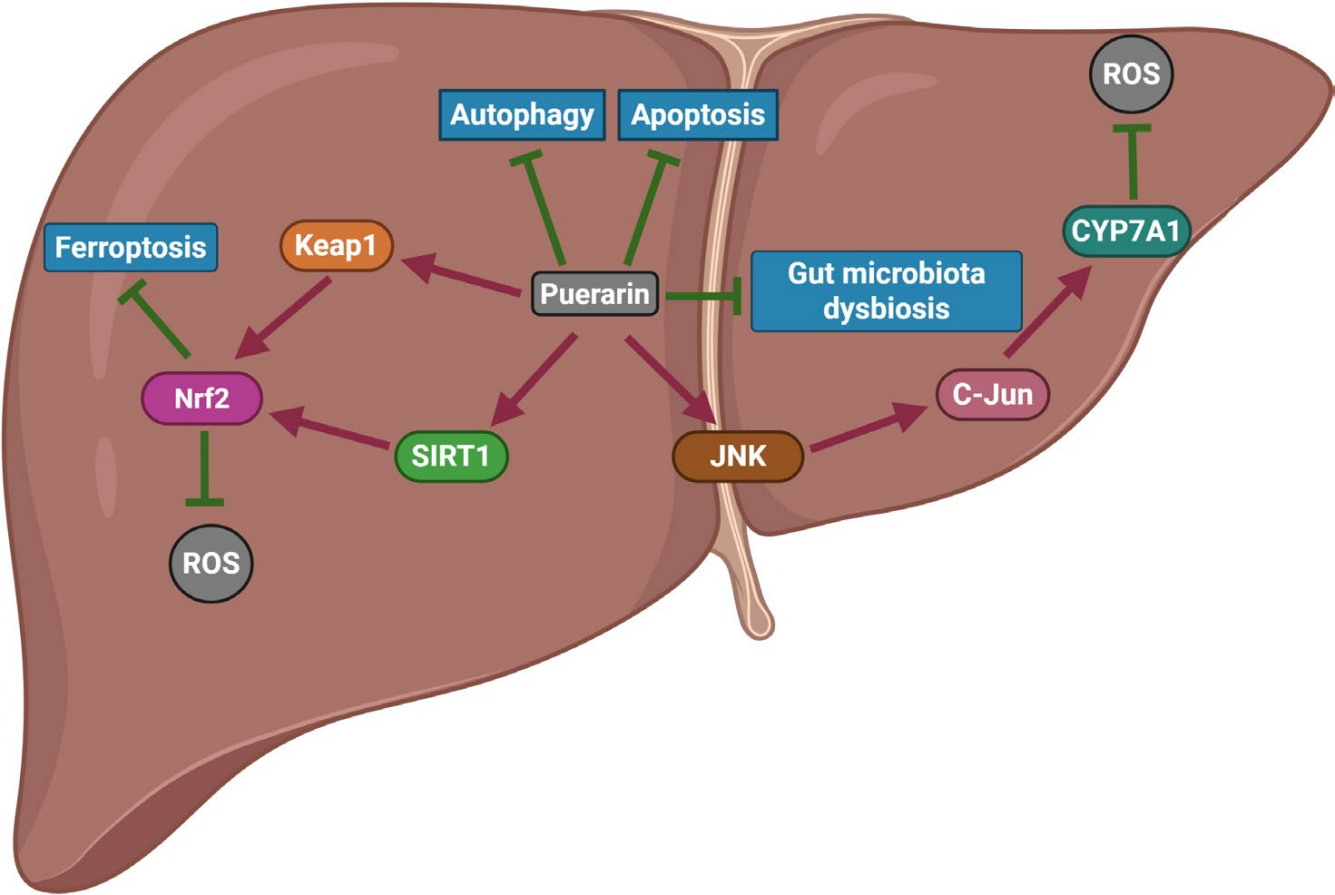
The role of puerarin in regulating other mechanisms.

In addition to lipid metabolism disorders and inflammation, puerarin also inhibits the development of NAFLD through other mechanisms (Figure 10). Oxidative stress is defined as an imbalance between oxidants and antioxidants. Under oxidative stress, excess reactive oxygen species (ROS) can destroy cell proteins, lipids, and DNA, leading to various pathologic conditions, such as aging, cancer, neurodegenerative diseases, cardiovascular diseases, diabetes, and so on. NAFLD is closely related to the presence of oxidative stress. Disruption of lipid metabolism causes lipid accumulation in the liver, which affects different ROS generators, including mitochondria, endoplasmic reticulum, and NADPH oxidase (Chen et al. 2020). At the same time, mitochondrial dysfunction is the main initiator of oxidative stress. They play a crucial role in the pathogenesis of NAFLD. At present, more and more studies have reported the antioxidant activity of puerarin in liver injury. In acetaminophen‐induced HepG2 cells, puerarin alleviated oxidative damage and mitochondrial dysfunction (Zhou, He, et al. 2023). In an acetaminophen‐induced mouse model, puerarin enhanced antioxidant activity by activating Keap1/Nrf2 signaling pathway (Zhou, He, et al. 2023). In CCl_4_‐induced mice, puerarin reduced the ROS accumulation of the liver via the JNK/c‐Jun/CYP7A1 pathway (Ma et al. 2014). Lead is a heavy metal that poses a major health problem to children. The existing epidemiological studies show that lead exposure was associated with an increased risk of NAFLD. Liu CM found that lead‐induced ROS accumulation and oxidative stress were suppressed by puerarin (Liu et al. 2011). These results suggest that puerarin may ameliorate NAFLD by inhibiting oxidative stress. Apoptosis is a basic biological phenomenon, which refers to the spontaneous death of cells in order to maintain homeostasis. Autophagy is also a basic biological phenomenon in which eukaryotic cells use lysosomes to degrade cytoplasmic proteins and damaged organelles. Both of them regulate the progression of NAFLD through multiple pathways. Zhou XL found that puerarin alleviated liver cell damage by inhibiting apoptosis and restoring autophagy activity (Zhou, Wan, et al. 2019). Ferroptosis, a form of programmed cell death discovered recently, plays a major role in NAFLD progression. Yang M found that puerarin ameliorates NAFLD by suppressing ferroptosis via the SIRT1/Nrf2 signaling pathway (Yang et al. 2023). Numerous studies have shown that NAFLD patients have altered gut microbiota. Gut microbiota influences the progression of NAFLD through its metabolites, bacterial endotoxins, and bile acids. Puerarin effectively ameliorated gut microbiota dysbiosis and modulated bile acid synthesis and transport (Yang et al. 2024). It has also been reported that puerarin combined with silymarin improved NAFLD by regulating gut microbiota (Wang, Jin, et al. 2024).

### 7.4 Puerarin and the Classical Signaling Pathways

**FIGURE 11:**
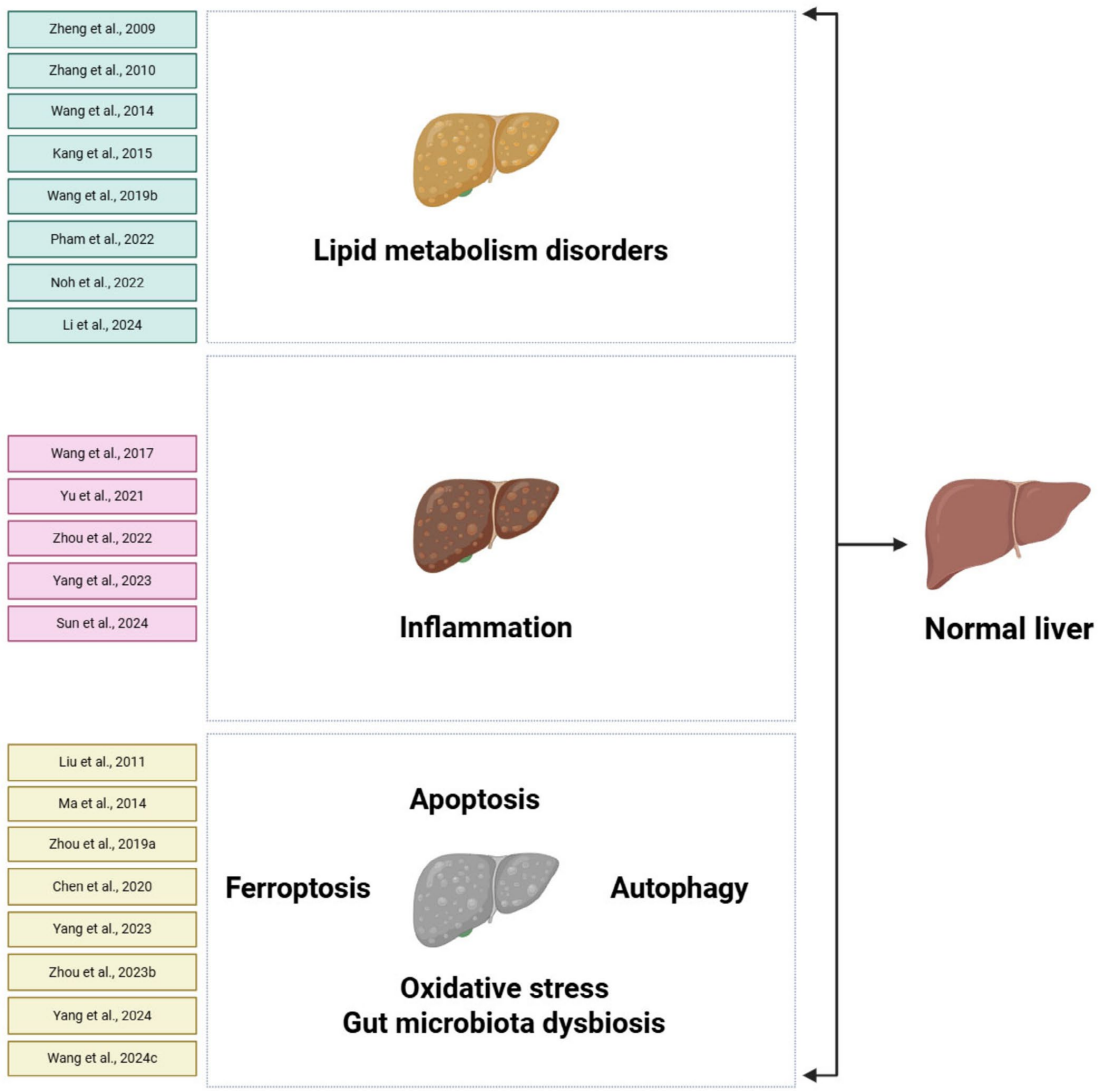
The bibliometric trends and the pharmacological mechanisms of puerarin in NAFLD.

At present, numerous studies have shown that puerarin can improve the progression of NAFLD through multiple signaling pathways (Figure 11). Among them, the AMPK, NF‐κB, and PI3K/AKT/mTOR signaling pathways are the most classic and valuable. As a core regulatory factor of energy metabolism, AMPK plays a significant role in glucose, lipid, and cholesterol metabolism. When the AMP/ATP ratio increases, AMPK is phosphorylated and activated by upstream kinases (LKB1 and CaMKKβ). Then, the activated AMPK phosphorylates downstream substrates, promoting catabolism (fatty acid oxidation, glycolysis) and inhibiting anabolism (fatty acid synthesis, cholesterol synthesis). In the pathological state of NAFLD, AMPK can phosphorylate SREBP‐1c and inhibit its nuclear translocation, down‐regulating the expression of FAS and ACC. Therefore, activating AMPK is a key strategy for improving lipid metabolism disorders in NAFLD. Current research indicates that puerarin activates AMPK, thereby reducing the expression of SREBP‐1 and ultimately inhibiting liver steatosis (Kang et al. 2015). NF‐κB signaling pathway is a very important signaling pathway, which is widely involved in many biological processes such as immune response, inflammatory response, apoptosis, and tumorigenesis. In the resting state, the NF‐κB p65/p50 dimer binds to IκBα and exists in the cytoplasm (an inactive form). When the cells are stimulated by pro‐inflammatory factors, oxidative stress or endotoxin, the IκB kinase (IKK) complex is activated, and then phosphorylates IκBα causing its degradation. Finally, the released NF‐κB translocates into the nucleus and initiates the transcription of inflammatory factors. These inflammatory cytokines further aggravate inflammation in the liver (Wang, Ou, et al. 2020). Puerarin can significantly inhibit the phosphorylation of IκBα, reduce the nuclear translocation of NF‐κB p65, and thereby decrease the expression of inflammatory factors (Yang et al. 2021). The PI3K/AKT/mTOR pathway is also a classic signaling pathway. When the PI3K is stimulated by growth factors and cytokines, it can activate the transforming growth factor‐β receptor (TGF‐βR) and the Vascular Endothelial Growth Factor Receptor (VEGFR), and convert Phosphatidylinositol 4,5‐bisphosphate (PIP2) into Phosphatidylinositol 3‐phosphate (PIP3). After PIP3 binds to the PH domain of AKT and 3‐Phosphoinositide‐dependent protein kinase 1 (PDK1), PDK1 can phosphorylate the Serc73 and Thr308 sites of AKT, thereby activating AKT. The activated AKT acts on the mammalian target of rapamycin (mTOR) and plays an important role in regulating liver lipid metabolism and insulin resistance. Studies have shown that puerarin improves the progression of NAFLD by activating the PI3K/AKT pathway (Wang, Yang, et al. 2019).

## 8 Discussion

Puerarin, a unique component of Pueraria, is also known as “phytoestrogen”. In recent years, puerarin has gradually become the focus of research in the natural products field. Bibliometrics is a discipline that uses mathematical and statistical methods to analyze relevant literature in a certain field and reveal its inherent laws and development trends. By evaluating and analyzing multiple dimensions such as the quantity, quality, citation, country, author, and institution of the literature, we can quickly understand the development status and trends in this field. Bibliometric analysis software includes CiteSpace and VOSviewer. Regrettably, there is currently no bibliometric analysis on puerarin, and we are unable to know the development trend and research hotspots of puerarin. Therefore, this study conducted a bibliometric analysis of the papers on puerarin based on the Web of Science core database. CiteSpace and VOSviewer software were used to analyze the article datasets in terms of the number of published articles, journal, national collaboration, institutional collaboration, author collaboration, co‐occurrence of keywords, and emergent keywords. Our results indicate that the overall trend of the number of published papers gradually increased over the years. By 2024, 209 papers have explored the puerarin. The 2004 articles include 9579 authors, 1721 institutions, 63 countries, 635 journals, and 7901 keywords. Among the authors, Wang L. has the largest number of published articles and the closest cooperation. Among the institutions, Beijing University of Chinese Medicine has the largest number of published papers. University of Chinese Academy of Sciences has the closest cooperation. Among the journals, Journal of ethnopharmacology has the largest number of published papers, and Biomedicine & pharmacotherapy has the closest cooperation. Among the keywords, the occurrence frequency and co‐occurrence intensity of puerarin are the highest. The bibliometric analysis reveals the global research landscape of puerarin. Due to the extensive application of pueraria lobata in traditional chinese medicine and the substantial investment of China in natural product research, China has an absolute advantage in publication output. The evolution of keyword clusters has progressed from analytical methods (high performance liquid chromatography) to mechanistic pathways (signaling pathway and gut microbiota). This transition indicates that the field has matured from characterizing the compound to elucidating its complex mechanisms, with the gut‐liver axis emerging as a novel frontier for future therapeutic development. In conclusion, more and more studies are exploring puerarin, and puerarin will remain a research hotspot in the future. Meanwhile, various cooperative network diagrams are in a concentrated state with high centrality, indicating that a good cooperative relationship has been formed in the current academic circle and scientific research forces are relatively concentrated.

This article has the following advantages: First, unlike previous bibliometric reviews that solely focus on publication trends, this study integrates bibliometric analysis with pharmacokinetics and toxicity, offering a multidimensional perspective for future therapeutic development. Second, this bibliometric study provides guidance for future treatment development. For instance, the emergence of “gut microbiota” as a distinct keyword cluster. This is particularly relevant for puerarin, given its poor oral bioavailability. Modulating the microbial community structure to reduce endotoxin production may provide new strategies for enhancing its efficacy and overcoming its pharmacokinetic limitations. This study also has some limitations: First, the literature included in this paper is limited to English journals, so some influential literature published in other languages may have been omitted. Second, we selected the WOS as the sole database for this bibliometric analysis due to its comprehensive coverage of high‐impact journals and its widespread use in bibliometric studies, which enhances the comparability and reproducibility of our results. Although Scopus, PubMed, and Embase also index relevant literature, the WOS provides robust citation data and standardized metadata, which is essential for co‐citation and collaboration analyses. However, we acknowledge that this approach may exclude some regional or interdisciplinary studies, which represents a limitation of this study. Third, VOSviewer software and CiteSpace software have many disadvantages. For example, some of the visualized data are in abbreviated form, and there may be errors when the data volume is too large.

With the rapid development of science, the effect of puerarin in the treatment of NAFLD has been clearly confirmed. Puerarin has abundant resources, low prices, strong pharmacological activity, and broad clinical application prospects. In the future, scientists should focus on improving its oral bioavailability and reducing its adverse reactions. At the same time, more research is needed to explore the mechanism of puerarin in the treatment of NAFLD.

## Author Contributions

**Wei Wang:** writing – original draft. **Xiang Liu:** writing – review and editing.

## Funding

The authors have nothing to report.

## Consent

All the authors give their consent for publication.

## Conflicts of Interest

The authors declare no conflicts of interest.

## Data Availability

The data that support the findings of this study are available from the corresponding author upon reasonable request.
